# Case Report: Juvenile Myelomonocytic Leukemia Underlying Ornithine Transcarbamylase Deficiency Safely Treated Using Hematopoietic Stem Cell Transplantation

**DOI:** 10.3389/fped.2022.898531

**Published:** 2022-05-06

**Authors:** Hiroi Eguchi, Toshihiko Kakiuchi, Masanori Nishi, Kanako Kojima-Ishii, Kei Nishiyama, Yuhki Koga, Muneaki Matsuo

**Affiliations:** ^1^Department of Pediatrics, Faculty of Medicine, Saga University, Saga, Japan; ^2^Department of Pediatrics, Graduate School of Medicine Sciences, Kyushu University, Fukuoka, Japan

**Keywords:** juvenile myelomonocytic leukemia, ornithine transcarbamylase deficiency, urea cycle disorder, hyperammonemia, hematopoietic stem cell transplantation, chemotherapy

## Abstract

**Background:**

Juvenile myelomonocytic leukemia (JMML), which is predominantly found in infants, is a clonal abnormality of pluripotent hematopoietic stem cells and presents with the symptoms of both myeloproliferative tumors and myelodysplastic syndromes. Estimates have shown that ~20 cases of JMML occur annually in Japan. Ornithine transcarbamylase deficiency (OTCD), the most common among all urea cycle disorders (UCDs), occurs in 1 of 80,000 people in Japan.

**Case Presentation:**

A 10-month-old infant who had fever, vomiting, and diarrhea for 2 days was referred to our hospital for the following abnormalities in blood tests: white blood cell count, 48,200/μL; hemoglobin, 9.0 g/dL; and platelet count, 135,000/μL. Bone marrow examination showed a nucleated cell count of 396,000/mm^3^ and blast cell count of 5.0%, as well as decreased mature granulocyte count and slightly myeloperoxidase stain-negative blasts but no monoclonal cell proliferation on May–Giemsa staining. Colony assay showed the proliferation of spontaneous colony and high sensitivity to granulocyte-macrophage colony-stimulating factor. Genetic analysis of peripheral blood mononuclear cells showed that the patient was positive for neuroblastoma RAS (*NRAS*) mutation. The patient was ultimately diagnosed with JMML. Approximately 170 days after his first hematopoietic stem cell transplantation (HSCT), the patient's JMML relapsed. Shortly after the recurrence, nausea, vomiting, hyperventilation, and decreased vitality were observed, followed by a decrease in the level of consciousness. The patient's ammonia level was 472 μmol/L. A test for seven different genetic mutations for the UCD showed the presence of c. 119G>A (amino acid change p. Arg40His). As such, late-onset OTCD was added to his diagnosis. Administration of sodium phenylacetate, l-arginine hydrochloride, and carnitine was continued following the diagnosis of OTCD, after which hyperammonemia was not observed. Regarding JMML relapse, HSCT was performed on day 405 after the first transplantation.

**Conclusion:**

Hyperammonemia should be considered a differential diagnosis when unexplained and non-specific symptoms occur during the treatment of hematologic malignancies. Patients should be tested for UCD as a cause of hyperammonemia, and treatment for hyperammonemia should be continued until the cause is identified. The patient shows normal developmental progress, has an intact neurological status, and has not experienced another hyperammonemia attack. His JMML has remained in remission for over 3 years.

## Introduction

Juvenile myelomonocytic leukemia (JMML) is a clonal abnormality of pluripotent hematopoietic stem cells, which is predominantly observed in infants and presents with the symptoms of both myeloproliferative tumors and myelodysplastic syndromes (1). Estimates have shown that approximately 20 cases of JMML occur annually in Japan, with the median age at diagnosis being 2 years (2). A suspected case of JMML typically presents with non-specific constitutional signs and symptoms, such as fever, infection, pallor, bleeding, cough, poor weight gain, maculopapular rash, lymphadenopathy, moderate hepatomegaly, marked splenomegaly, leukocytosis, absolute monocytosis, anemia, and thrombocytopenia (3).

Ornithine transcarbamylase deficiency (OTCD) is an X-linked genetic disorder that prevents the breakdown and excretion of ammonia. This allows ammonia to attain toxic levels, which affect the central nervous system (4). OTCD is the most common among all urea cycle disorders (UCDs) (5), and occurs in 1 of 80,000 people in Japan (6, 7). Male neonates with OTCD often experience ammonia toxicity, protein intolerance, and die within a week after birth. However, those with late-onset OTCD (age at onset, ≥28 days) present with a wide range of symptoms that range from asymptomatic phenotype to hyperammonemia, coma, and death (8, 9).

To the best of our knowledge, there has been no report on a case presenting with both JMML and OTCD. Herein, we report the case of an infant boy diagnosed with late-onset OTCD who developed hyperammonemia at relapse after hematopoietic stem cell transplantation (HSCT) for JMML.

## Case Presentation

**Figure 1:**
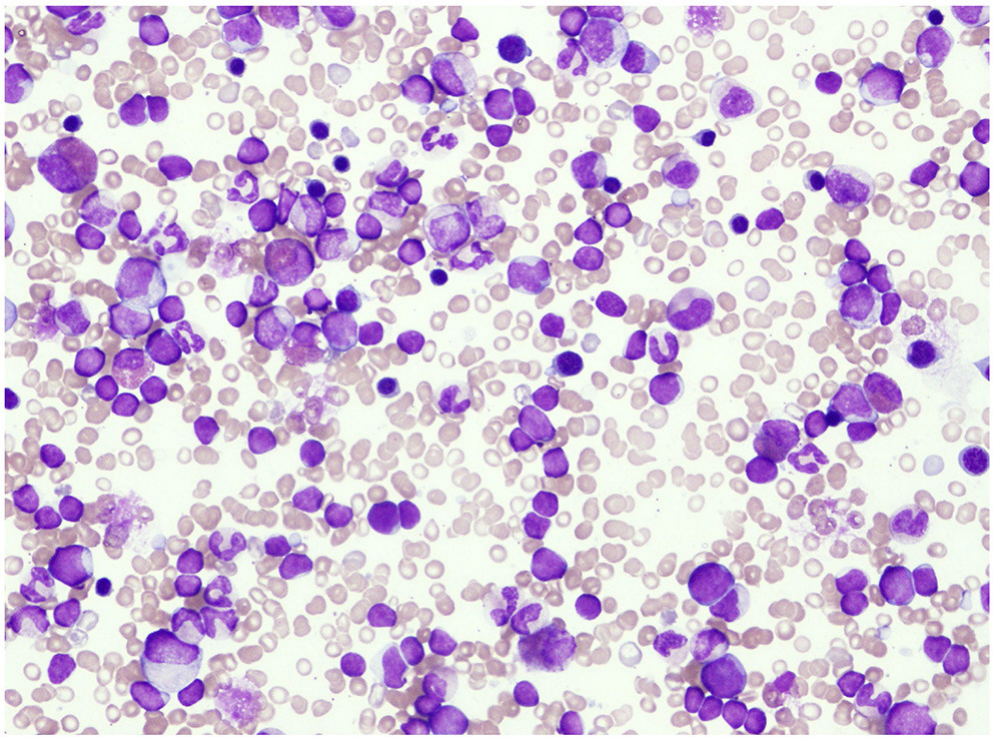
Bone marrow examination showed a decrease in mature granulocytes and slight myeloperoxidase stain-negative blast cells but no monoclonal cell proliferation with May–Giemsa staining.

A 10-month-old infant who had fever, vomiting, and diarrhea for 2 days was referred to our hospital owing to blood test abnormalities. He had no family history of OTCD. He had neither abnormal eating habits nor poor appetite nor insidious symptoms such as liver disorders, behavioral disorders, psychiatric symptoms, growth delay, and developmental delay. His laboratory data were as follows: white blood cell count (WBC), 48,200/μL [normal range (NR): 7,000–15,000/μL]; hemoglobin count, 9.0 g/dL (NR: 13.5–18.0 g/dL); platelet count, 135,000/μL (NR: 150,000–330,000/μL); and C-reactive protein content, 10.3 mg/dL (NR: <0.3 mg/dL). The patient had a height of 69.5 cm [−1.8 standard deviation (SD)] and a weight of 8,800 g (−0.4 SD). His vital signs showed an abnormal body temperature of 39.4°C and a heart rate of 164 beats/min. Physical examination revealed pigmented papules scattered over his extremities, a liver palpable 5 cm in the right hypochondriac region, and a spleen palpable 6 cm in the left hypochondriac region. The initial laboratory tests conducted at our facility showed the following results: WBC count, 60,900/μL; neutrophilic rate, 37.7%; lymphocyte rate, 42.7%; monocyte rate, 19.1% (3–9%); hemoglobin count, 9.1 g/dL; platelet count, 145,000/μL; fetal hemoglobin level, 19.1% (NR: <1.2%); aspartic aminotransferase level, 63 IU/L (NR: 20–45 IU/L); alanine aminotransferase level, 36 IU/L (NR: 4–24 IU/L); gamma-glutamyl transpeptidase level, 40 IU/L (NR: 5–17 IU/L); and ammonia level, 83 μg/dL (NR: <80 μg/dL). Bone marrow examination showed a nucleated cell count of 396,000/mm^3^, megakaryocyte count of 0/mm^3^, monocyte rate of 5.4%, blast cell count of 5.0%, and myeloid to erythroid ratio of 3.41. It also showed decreased mature granulocytes and count and slightly myeloperoxidase stain-negative blast cells but no monoclonal cell proliferation using May–Giemsa staining (Figure 1). Colony assay showed spontaneous colony proliferation and high sensitivity to granulocyte-macrophage colony-stimulating factor. Genetic analysis of peripheral blood mononuclear cells showed that the patient was positive for neuroblastoma RAS (*NRAS*) mutation [Codon 12, 13 mutation-positive c.35G>A (amino acid change p.G12D)]. No germline mutation of *NRAS* was observed. Chromosome analysis showed a normal karyotype. Based on these findings, the patient was diagnosed with JMML.

**Figure 2:**
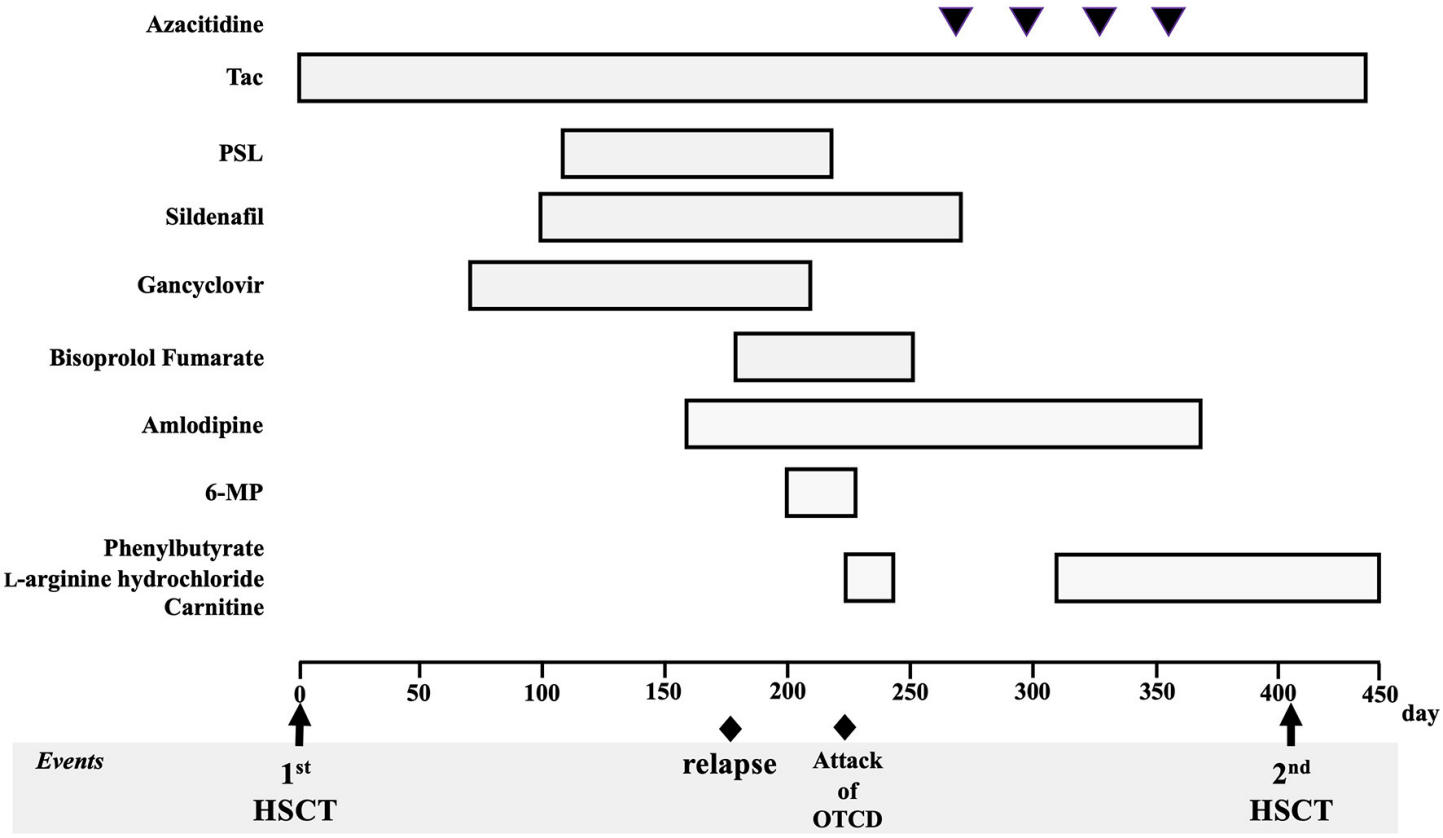
Patient's clinical course and treatment for juvenile myelomonocytic leukemia and complications. Tac, tacrolimus; PSL, prednisolone; 6-MP, 6-mercaptopurine; HSCT, hematopoietic stem cell transplantation; OTCD, ornithine transcarbamylase deficiency.

Figure 2 shows the clinical course of the patient. He had never been treated with any cytoreductive therapy before the first HSCT. After being transferred to an HSCT facility, he was pretreated with busulfan (1.2 mg/m^2^ for 4 days), fludarabine (30 mg/m^2^ for 4 days), and melphalan (90 mg/m^2^ for 2 days) and underwent an allogeneic cord blood transplantation (CBT) 8 months after the diagnosis of JMML. The acute clinical course after CBT was unremarkable, and the complete chimera was confirmed on day 25. On day 66, cytomegalovirus pneumonia was observed; the patient was treated with ganciclovir, and he showed a mild recovery. On day 87, pulmonary hypertension was noted, which was treated with steroids and sildenafil. On day 153, hypertension was noted, and the patient was subsequently treated with antihypertensive drugs. On day 167, the patient suffered from tubulopathy and nephrotic syndrome, but he showed mild recovery after hyperbaric medicine treatment. Throughout this course, ammonia levels were normal after several measurements.

On approximately day 170, hematopenia (WBC, 2,600/μL; hemoglobin, 11.0 g/dL; and platelet count, 69,000/μL) appeared gradually, and on day 179, a positive peripheral blood mononuclear cell *NRAS* mutation was confirmed, which appeared to have relapsed. Tacrolimus, which was used for managing various complications that could not be ruled out as graft-versus-host disease, was difficult to discontinue. Mercaptopurine hydrate (50 mg/m^2^) was then started; however, immediately following administration, nausea, vomiting, hyperventilation, and decreased vitality were observed, followed by a decrease in the level of consciousness. At the start of the medication, his WBC count was 2,300/μL, and chimerism at this time was not confirmed. The ammonia level on the 10th day after the onset of these symptoms was 472 μmol/L and carbon dioxide partial pressure decreased with respiratory alkalosis (pCO_2_ 23.8 mmHg), which was determined to have been caused by hyperammonemia. Administration of sodium phenylacetate (250 mg/kg), l-arginine hydrochloride (400 mg/kg), and sodium benzoate (250 mg/kg) was then initiated. On day 222 (19 days after the start of the medication), mercaptopurine hydrate was discontinued. Considering his extremely high ammonia levels, we also began preparations for hemodialysis. However, his ammonia levels quickly dropped and normalized (115 μmol/L at 12 h, 70 μmol/L at 24 h after the initiation of nitrogen scavenger therapy), accompanied by the rapid disappearance of all symptoms. At the onset of the hyperammonemia attack, the nephrotic syndrome persisted; therefore, potassium intake was restricted due to poor potassium excretion caused by tubular damage. The nitrogen scavenger therapy was gradually reduced after referring to the ammonia levels and blood amino acid analysis results and was finally stopped. No neurological sequelae were observed. Plasma amino acid analysis showed high glutamate levels and normal citrulline levels, with urinary organic acid analysis showing the presence of high orotic acid levels. A test for seven different genetic mutations for the UCD showed ornithine transcarbamylase (*OTC*) gene mutation [c. 119G>A, (amino acid change p. Arg40His)]. Thereafter, late-onset OTCD was added to his diagnosis. None of his blood relatives had UCD and he did not present with any symptoms of suspected hyperammonemia. Administration of sodium phenylbutyrate, l-arginine hydrochloride, and carnitine was resumed after the diagnosis of OTCD, and no hyperammonemia was observed. Four cycles of azacytidine (75 mg/m^2^ for 3 days) were administered for the bridging therapy before HSCT. The ethical aspects of the use of azacytidine were reviewed and approved by the institutional review board of Saga University Hospital (approval date: September 3, 2018).

Regarding the relapse of JMML, he was pretreated with fludarabine (30 mg/m^2^ for 5 days) and melphalan (90 mg/m^2^ for 2 days). Subsequently, he underwent unrelated allogeneic bone marrow transplantation on day 405 after the first transplant and on day 165 after the onset of hyperammonemia. Administration of the nitrogen scavenger agents, sodium phenylacetate (250 mg/kg) and l-arginine hydrochloride (125 mg/kg), was resumed in time with the second HSCT (immediately after the genetic diagnosis of OTCD), and HSCT was completed safely without inducing another hyperammonemia attack. We introduced a protein intake limit of 1.5 g per body weight per day. The complete chimera was confirmed on day 29. He shows normal developmental progress, with an intact neurological status, and has not experienced another hyperammonemia attack. Furthermore, he has no abnormal eating habits. His JMML has remained in remission for over 3 years.

## Discussion

The present case presents an extremely rare combination of JMML and OTCD. One noteworthy aspect of the present case is the proven involvement of congenital UCD in the pathogenesis of hyperammonemia associated with cancer treatment. The incidence of JMML and OTCD is 1–2/million and 1/80,000, respectively (6, 7, 10), which suggests the extremely low frequency of complicated cases. *OTC* is present at Xp21, whereas *NRAS* is present at 1p13.2. Considering that these two diseases have unrelated genetic mutations, the probability of encountering a case with a combination of JMML and OTCD, as in the present case, was astronomically low. Despite including not only JMML but also hematologic oncology diseases and chemotherapy-related cases in our literature search, we found no reports on hyperammonemia with underlying OTCD.

Chemotherapy-related hyperammonemia was first reported in the 1980s (11, 12), followed by cases occurring after HSCT (13). In most cases, no obvious cause was identified, and the disease was reported as idiopathic hyperammonemia. We have no knowledge regarding any report demonstrating the involvement of an inherited metabolic disease, as observed in the present case. Although there has been a report of those associated with 5-fluorouracil, ammonia is a known metabolite of 5-fluorouracil, with evidence showing a transient and favorable course (14). Only one case has been reported, in which the disease developed during the early steroid treatment phase of acute lymphocytic leukemia (15). No previous reports have suggested an association between hyperammonemia and mercaptopurine, which is the most recently used chemotherapy drug in the present case. There is an exceptional report of a case of acquired carbamoylphosphate synthetase 1 deficiency following transplantation in which the pathogenesis was presumed (16). Chemotherapy-associated idiopathic hyperammonemia may be a multifactorial condition with various onset times, causative agents, and underlying diseases. However, as noted in the present case, UCD should also be considered. Symptoms of hyperammonemia are non-specific and include nausea, vomiting, lack of vigor, seizures, and impaired consciousness, which are relatively frequently encountered during chemotherapy. When abnormal neurological findings are observed, differentiating them from those caused as a result of the disease treatment should be a priority, including direct central damage by anticancer drugs, central nervous system metastasis of malignancy, thrombosis, and hemorrhage, although measuring ammonia levels has also been considered important. In addition, if hyperammonemia is observed, even during chemotherapy, UCD should be considered a potential cause. Therefore, clinicians should have a detailed knowledge of the presenting signs and history of the patient to suspect UCD (17).

OTCD is known to be triggered by starvation, infection, fever, vomiting, excessive protein intake, gastrointestinal bleeding, excess glucocorticoids, and various drugs (18, 19). The severity of male OTCD is correlated with the deleterious mutation that results in structural changes in the OTC protein. In a Japanese nationwide study, c.119G>A of *OTC* mutation was found to be the most common variant and was observed in male patients with late-onset OTCD (20). The timing of the hyperammonemia attacks in the patient was consistent with that observed in the previous reports. In the present case, the patient had good tolerance to various drugs (ganciclovir, tacrolimus, azacytidine, fludarabine, melphalan, and busulfan). It is noteworthy that some of the medications used in the patients included high-dose chemotherapy. High-dose chemotherapy during both transplantations did not trigger hyperammonemic attacks associated with OTCD, suggesting that the intensity of chemotherapy was not necessarily the only factor involved in the development of the disease. We found no reports linking mercaptopurine to hyperammonemic attacks associated with UCDs. However, mercaptopurine most likely triggers hyperammonemic attacks. In our case, this drug was administered for disease control during recurrence; it may have caused tumor collapse, which consequently served as a source of nitrogen. We hypothesized that tumor collapse alone provided a sufficient source of nitrogen to cause the hyperammonemic attack. However, this hypothesis hardly explains the entire course of our patient's hyperammonemic attack, and no facts can support specific evidence for other causal hypotheses. For example, allopurinol, which indirectly affects the urea cycle, was not administered. The patients' protein intake was approximately 1.5 g/kg/day immediately before the hyperammonemic attack onset. Moreover, the patient did not receive parenteral nutrition. Therefore, mercaptopurine is a highly suspected agent because of its temporal anteroposterior relationship with hyperammonemic attacks. Unfortunately, neither an association nor a pathophysiologically plausible hypothesis could be found in known reports. The onset of OTCD occurred at a time when the child had multiple uncontrollable problems, including post-transplantation relapse and various post-transplantation complications. Davies et al. reported that the post-transplantation onset of idiopathic hyperammonemia occurred at a median onset time of 25 days, with the latest onset occurring at 106 days (13). It can be argued that post-transplantation hyperammonemia, although common during the acute post-transplantation period, is not necessarily unique to the acute period. The timing of the onset of the hyperammonemia attack in the current case was as late as day 223 after the first transplantation. Post-transplantation management requires long-term follow-up, including hyperammonemia. Mitchell et al. reported that postmortem examination of the liver in five patients failed to reveal ultrastructural evidence characteristic of the Reye's syndrome (11). In addition, Frere et al. hypothesized the involvement of gastrointestinal hemorrhage and infection as pathological causes of hyperammonemia after HSCT (21). However, this hypothesis is not strong, and these conditions are not likely to be associated with the induction of seizures in our patient's case. Almost all published studies concluded that post-HSCT hyperammonemia is idiopathic (22). In addition, the present case showed that the second transplantation could be safely managed without acute attacks of OTCD by introducing the nitrogen removal treatment in advance. If hyperammonemia is observed during chemotherapy or after transplantation, we believe that continuing the treatment for hyperammonemia until and after identifying the cause is imperative because many deaths have been reported in several studies regarding unexpected hyperammonemia during chemotherapy (11–13). The clinical outcome of our patient was excellent, and he is currently showing normal developmental range, with an intact neurological status and has not experienced another hyperammonemia attack. His JMML has remained in remission for over 3 years. Kido et al. reported that patients with UCD with a maximum ammonia concentration of >360 μmol/L either died (15%, 11/74) or developed mental retardation (51%, 38/74) (23). Fortunately, our patient is doing well at present and has no neurological sequelae, although his maximum ammonia concentration was above 360 μmol/L.

In conclusion, hyperammonemia should be considered as a differential diagnosis when unexplained loss of consciousness and repeated vomiting occur during hematologic malignancy treatment. Patients should be tested for UCD as a cause of hyperammonemia even under the special circumstances of being on chemotherapy. The present case suggests that even when OTCD diagnosis is confirmed, overcoming highly invasive treatments, such as hematopoietic stem cell transplantation, is possible through adequate preparation and continued administration of hyperammonemic drugs.

## Data Availability Statement

The original contributions presented in the study are included in the article/supplementary material, further inquiries can be directed to the corresponding author.

## Ethics Statement

Ethical review and approval was not required for the study on human participants in accordance with the local legislation and institutional requirements. Written informed consent to participate in this study was provided by the participants' legal guardian/next of kin.

## Author Contributions

HE was involved in patient care as well as the drafting, review, and revision of the initial manuscript. TK, KK-I, and KN was involved in the patient's treatment decision as well as the review and revision of the initial manuscript. MN was involved in patient care as well as the review and revision of the initial manuscript. YK was involved in the patient's treatment decision and project administration, as well as the review and revision of the initial manuscript. MM was involved in patient care and project administration, as well as the review and revision of the initial manuscript. All authors have approved the final manuscript submission and have agreed to be accountable for all aspects of the study.

## Conflict of Interest

The authors declare that the research was conducted in the absence of any commercial or financial relationships that could be construed as a potential conflict of interest.

## Publisher's Note

All claims expressed in this article are solely those of the authors and do not necessarily represent those of their affiliated organizations, or those of the publisher, the editors and the reviewers. Any product that may be evaluated in this article, or claim that may be made by its manufacturer, is not guaranteed or endorsed by the publisher.

## References

[1] Vardiman JW Thiele J Arber DA Brunning RD Borowitz MJ Porwit A. The 2008 revision of the World Health Organization (WHO) classification of myeloid neoplasms and acute leukemia: rationale and important changes. Blood. (2009) 114:937–51. 10.1182/blood-2009-03-209262 19357394

[2] Niemeyer CM Arico M Basso G Biondi A Cantu Rajnoldi A Creutzig U. Chronic myelomonocytic leukemia in childhood: a retrospective analysis of 110 cases. European Working Group on myelodysplastic Syndromes in Childhood (EWOG-MDS). Blood. (1997) 89:3534–43 9160658

[3] Puri K Singh P Das RR Seth R Gupta R. Diagnostic dilemma of JMML coexisting with CMV infection. Indian J Pediatr. (2011) 78:485–7. 10.1007/s12098-010-0355-z 21193971

[4] Donovan K Guzman N. Ornithine transcarbamylase deficiency. StatPearls, StatPearls Publishing LLC. (2022). Treasure Island (FL): StatPearls Publishing Copyright © 2022 30725942

[5] Choi JH Lee BH Kim JH Kim GH Kim YM Cho J. Clinical outcomes and the mutation spectrum of the OTC gene in patients with ornithine transcarbamylase deficiency. J Hum Genet. (2015) 60:501–7. 10.1038/jhg.2015.54 25994866

[6] Nagata N Matsuda I Matsuura T Oyanagi K Tada K Narisawa K. Retrospective survey of urea cycle disorders: Part 2. Neurological outcome in forty-nine Japanese patients with urea cycle enzymopathies. Am J Med Genet. (1991) 40:477–81. 10.1002/ajmg.1320400421 1746614

[7] Nagata N Matsuda I Oyanagi K. Estimated frequency of urea cycle enzymopathies in Japan. Am J Med Genet. (1991) 39:228–9. 10.1002/ajmg.1320390226 2063931

[8] Fujisawa D Mitsubuchi H Matsumoto S Iwai M Nakamura K Hoshide R. Early intervention for late-onset ornithine transcarbamylase deficiency. Pediatr Int. (2015) 57:e1–3. 10.1111/ped.12457 25711267

[9] Ausems MG Bakker E Berger R Duran M van Diggelen OP Keulemans JL. Asymptomatic and late-onset ornithine transcarbamylase deficiency caused by a A208T mutation: clinical, biochemical and DNA analyses in a four-generation family. Am J Med Genet. (1997) 68:236–9. 9028466

[10] Chan RJ Cooper T Kratz CP Weiss B Loh ML. Juvenile myelomonocytic leukemia: a report from the 2nd International JMML Symposium. Leuk Res. (2009) 33:355–62. 10.1016/j.leukres.2008.08.022 18954903 PMC2692866

[11] Mitchell RB Wagner JE Karp JE Watson AJ Brusilow SW Przepiorka D. Syndrome of idiopathic hyperammonemia after high-dose chemotherapy: review of nine cases. Am J Med. (1988) 85:662–7. 10.1016/S0002-9343(88)80239-0 3189370

[12] Watson AJ Chambers T Karp JE Risch VR Walker WG Brusilow SW. Transient idiopathic hyperammonaemia in adults. Lancet. (1985) 2:1271–4. 10.1016/S0140-6736(85)91554-5 2866337

[13] Davies SM Szabo E Wagner JE Ramsay NK Weisdorf DJ. Idiopathic hyperammonemia: a frequently lethal complication of bone marrow transplantation. Bone Marrow Transplant. (1996) 17:1119–25 8807124

[14] Liaw CC Wang HM Wang CH Yang TS Chen JS Chang HK. Risk of transient hyperammonemic encephalopathy in cancer patients who received continuous infusion of 5-fluorouracil with the complication of dehydration and infection. Anti Cancer Drugs. (1999) 10:275–81. 10.1097/00001813-199903000-00004 10327032

[15] Kobayashi S Ito M Sano H Mochizuki K Akaihata M Waragai T. Idiopathic hyperammonemia that developed during initial treatment with steroid in a patient with newly diagnosed leukemia. J Pediatr Hematol Oncol. (2015) 37:e361–3. 10.1097/MPH.0000000000000255 25222063

[16] Laemmle A Hahn D Hu L Rüfenacht V Gautschi M Leibundgut K. Fatal hyperammonemia and carbamoyl phosphate synthetase 1 (CPS1) deficiency following high-dose chemotherapy and autologous hematopoietic stem cell transplantation. Mol Genet Metab. (2015) 114:438–44. 10.1016/j.ymgme.2015.01.002 25639153

[17] Kido J Matsumoto S Häberle J Nakajima Y Wada Y Mochizuki N. Long-term outcome of urea cycle disorders: report from a nationwide study in Japan. J Inherit Metab Dis. (2021) 44:826–37. 10.1002/jimd.12384 33840128

[18] Gascon-Bayarri J Campdelacreu J Estela J Reñé R. Severe hyperammonemia in late-onset ornithine transcarbamylase deficiency triggered by steroid administration. Case Rep Neurol Med. (2015) 2015:453752. 10.1155/2015/453752 25949836 PMC4407407

[19] Helman G Pacheco-Colón I Gropman AL. The urea cycle disorders. Semin Neurol. (2014) 34:341–9. 10.1055/s-0034-1386771 25192511

[20] Kido J Matsumoto S Sugawara K Sawada T Nakamura K. Variants associated with urea cycle disorders in Japanese patients: nationwide study and literature review. Am J Med Genet A. (2021) 185:2026–36. 10.1002/ajmg.a.62199 33851512

[21] Frere P Canivet JL Gennigens C Rebeix JP Fillet G Beguin Y. Hyperammonemia after high-dose chemotherapy and stem cell transplantation. Bone Marrow Transplant. (2000) 26:343–5. 10.1038/sj.bmt.1702485 10967577

[22] Tse N Cederbaum S Glaspy JA. Hyperammonemia following allogeneic bone marrow transplantation. Am J Hematol. (1991) 38:140–1. 10.1002/ajh.2830380213 1951305

[23] Kido J Nakamura K Mitsubuchi H Ohura T Takayanagi M Matsuo M. Long-term outcome and intervention of urea cycle disorders in Japan. J Inherit Metab Dis. (2012) 35:777–85. 10.1007/s10545-011-9427-0 22167275

